# Necrology

**Published:** 1902-01

**Authors:** 


### NECROLOGY.

THOMAS F. BARRON, V.S., died at Baltimore, Md., on November 20, 1901. This well-known veterinarian passed away at the age of fifty-nine years, after an acute attack of gastritis, which was followed by a greatly depressed heart action and death. He had not enjoyed vigorous health for several years, but his death was very unexpected during his last illness.

Dr. Barron had spent his whole life in the pursuit of veterinary medicine, having succeeded to his father's extensive practice, and was an earnest student, a devoted reader of veterinary literature, and had surrounded himself with one of the largest and most comprehensive veterinary libraries of the land. He filled an enviable position among a large clientage.

He took an active part in association work in his own State, being one of the first members of the Maryland State Veterinary Medical Association. He joined the American Veterinary Medical Association in 1887 and attended many of its meetings.

Somewhat reserved in manner, but a firm and devoted friend when once you had gained his good-will. Honored and respected by his colleagues in his native State, the following resolutions were adopted as a minute of the

MARYLAND STATE VETERINARY MEDICAL SOCIETY.

"Inasmuch as Divine Providence has removed from our midst our late associate, Dr. Thomas F. Barron, one of the pioneers of the veterinary profession in Maryland, we desire to record the deep sense of our loss of the gentlemanly attributes and the proficient professional ability possessed by him and accorded to all; and, further, to express to the one who mourns most deeply his decease our sincere sympathy and condolence."

AT the Episcopal Hospital in Philadelphia, on December 21, 1901, Dr. James Beatty died of purpura hemorrhagica, after about two weeks' illness.

Dr. Beatty was born in Philadelphia, December 21, 1859, and was just forty-two years old at the time of his death. He graduated at the Veterinary Department of the University of Pennsylvania in 1898. Soon after his graduation he was employed by the Government as a meat inspector. His first assignment under the Bureau of Animal Industry was in one of the Western dressed beef centres, but for the past two years he was stationed in Philadelphia. He was a life member of Orient Lodge, No. 289, F. and A. M., and had taken nearly all the degrees in Freemasonry. Of a genial disposition, he made many friends. C. J. M.

